# Depression, anxiety and stress in dental students

**DOI:** 10.5116/ijme.5910.b961

**Published:** 2017-05-24

**Authors:** Sumaya Basudan, Najla Binanzan, Aseel Alhassan

**Affiliations:** 1 Department of Restorative Dental Sciences, College of Dentistry, King Saud University, Saudi Arabia; 2 Endodontic Postgraduate Program at the College of Dentistry, King Saud University, Saudi Arabia; 3 Saudi Board of Restorative Dentistry, Saudi Arabia

**Keywords:** Keywords: Depression, anxiety, stress, dental student, psychological health

## Abstract

**Objectives:**

To measure the occurrence and levels of depression, anxiety and stress in undergraduate dental students using the Depression, Anxiety and Stress Scale (DASS-21).

**Methods:**

This cross-sectional study was conducted in November and December of 2014. A total of 289 dental students were invited to participate, and 277 responded, resulting in a response rate of 96%. The final sample included 247 participants. Eligible participants were surveyed via a self-reported questionnaire that included the validated DASS-21 scale as the assessment tool and questions about demographic characteristics and methods for managing stress.

**Results:**

Abnormal levels of depression, anxiety and stress were identified in 55.9%, 66.8% and 54.7% of the study participants, respectively. A multiple linear regression analysis revealed multiple predictors: gender (for anxiety b=-3.589, p=.016 and stress b=-4.099, p=.008), satisfaction with faculty relationships (for depression b=-2.318, p=.007; anxiety b=-2.213, p=.004; and stress b=-2.854, p<.001), satisfaction with peer relationships (for depression b=-3.527, p<.001; anxiety b=-2.213, p=.004; and stress b=-2.854, p<.001), and dentistry as the first choice for field of study (for stress b=-2.648, p=.045). The standardized coefficients demonstrated the relationship and strength of the predictors for each subscale. To cope with stress, students engaged in various activities such as reading, watching television and seeking emotional support from others.

**Conclusions:**

The high occurrence of depression, anxiety and stress among dental students highlights the importance of providing support programs and implementing preventive measures to help students, particularly those who are most susceptible to higher levels of these psychological conditions.

## Introduction

Dental education can be a significant source of stress among dental students, and studies have observed higher levels of stress among dental students than in the general population.^1^ A large body of literature examining stress in undergraduate dental students has revealed a significant increase in stress that intensifies with students’ year of study.^2^ Stress is a result of certain external physical or mental factors that affect an individual’s physical and psychological well-being.^3^

In their systematic review, Alzahem and colleagues^4^ categorized stressors into five groups: living accommodation, educational environment, and personal, academic and clinical factors. Examinations and grades are frequently reported to be the most stressful factors, along with limited time for relaxation.^4^^,^^5^ Additionally, research has found that stressors are not globally equivalent and are influenced by local and cultural factors.^4^ In Saudi Arabia, a number of studies have assessed levels of stress among dental students and related stressors, and the most commonly cited stressors are gender, year of study, marital status, first choice of admission, financial problems, living arrangement, examinations and grades, workload, and patients.^6^^-^^10^

Long-term stress has been linked to the development of other disorders. A study conducted in a Swedish population found an association between different stress levels and mental health: high levels of stress were associated with depression, whereas low and moderate stress levels were associated with anxiety.^11^ Moreover, Alansari^12^ surveyed over 9000 undergraduate students in 18 countries and found depression to be positively and significantly correlated with anxiety. Anxiety is associated with autonomic arousal, skeletal muscle effects, and subjective experience of an anxious affect, and it is defined by the American Psychological Association as an "emotion characterized by feelings of tension, worried thoughts and physical changes such as increased blood pressure".^13^ The World Health Organization defines depression as "a common mental disorder, characterized by sadness, loss of interest or pleasure, feelings of guilt or low self-worth, disturbed sleep or appetite and feelings of tiredness and poor concentration. It can be long-lasting or recurrent, substantially impairing a person’s ability to function at work, school or cope with daily life".^14^

Despite their interrelationship, anxiety and depression in dental students have not been explored as frequently as stress. Previous studies have implemented specific tools designed to evaluate the construct of stress only, such as the commonly used Dental Environmental Stress (DES) Scale.^15^ To assess psychological well-being, tools such as the Beck Depression Inventory (BDI),^16^^,^^17^ the Maslach Burnout Inventory,^18^ the Cambridge Depersonalization Scale,^19^ the Hospital Anxiety and Depression Scale,^19^ the Spielberger State-Trait Anxiety Inventory,^17^ and the Self-Rating Depression Scale^20^ have been applied independently or in combination. Thus, it would be beneficial to examine depression, anxiety, and stress using one tool rather than multiple assessments. The Depression, Anxiety and Stress Scale (DASS-21) measures the three dimensions of these psychological conditions in a single, concise and comprehensive scale. To the best of our knowledge, this tool has not been applied to measure these psychological conditions exclusively in undergraduate dental students.

Although several studies have assessed levels of stress and identified stressors in the dental environment in Saudi Arabia, no studies have used measures of depression and anxiety to assess the psychological well-being of undergraduate dental students specifically. Therefore, our aim was to measure the levels of depression, anxiety and stress in dental students at King Saud University (KSU) using the DASS-21 and to investigate the relationship between these levels and previously reported stressors.

## Methods

### Study design and participants

This cross-sectional study was conducted at the College of Dentistry of KSU in Riyadh City, Saudi Arabia, during November and December of 2014. All undergraduate dental students were eligible to participate. First-year dental students who had not yet completed one year of dental school and students receiving any psychological management (cognitive or behavioral therapy, medication or a combination; whose responses could be biased due to the effect of treatment on their psychological status) were excluded from the study. Ethical approval was obtained from the Ethics Committee of the College of Dentistry Research Center (CDRC) at KSU (CDRC NO: IR0096).

In total, 289 of 595 students from all study years were invited to participate.  The questionnaire was completed by 277 students, resulting in a response rate of 95.8%. Of the surveyed participants, 30 (10.83%) disclosed receiving psychological management and were excluded from the study; thus, the final sample size was 247 participants. The demographic characteristics of the study participants are presented in Table 1.

**Table 1:** Demographics of the sample population

%	No.	Demographic variable	
54.2	134	Male	Gender
45.7	113	Female	
89.1	220	Single	Marital status
8.9	22	Married	
0	0	Widowed	
2	5	Divorced	
19	47	2^nd^ year	Year of study
21.4	53	3^rd^ year	
19.4	48	4^th^ year	
19.0	47	5^th^ year	
21	52	Interns	
13.8	34	2.75 - < 3.75	GPA (of previous year)
44.1	109	3.75 - < 4.5	
42.1	104	4.5 - 5	
36.4	90	No	Dentistry 1^st^ choice
63.5	157	Yes	
83.0	205	No	Financial responsibilities
17.0	42	Yes	
79.8	197	Satisfied	Satisfaction with peer relationships
7.3	18	Dissatisfied	
12.9	32	Neither	
60.3	149	Satisfied	Satisfaction with faculty relationships
19.4	48	Dissatisfied	
20.2	50	Neither	
51.8	128	Satisfied	Satisfaction with college overall
34.0	84	Dissatisfied	
14.2	35	Neither	

### Study tool

A two-part, self-administered questionnaire was developed in English. The first section included 11 questions related to demographic characteristics such as gender, marital status, year of study, grade point average (GPA), and whether dental school was the student’s first admission choice. Subsequently, the students answered questions scored on a Likert scale (from 0 to 5) assessing their satisfaction with their peer and faculty relationships and the academic environment in general. A final question addressed the students’ stress coping methods (more than one option could be selected). The second section of the questionnaire contained the 21 items from the short-form version of the DASS.

The DASS was developed by Lovibond and Lovibond^21^ to assess the core symptoms of depression, anxiety and stress and has also been used to evaluate patients’ response to treatment.^21^ The DASS has demonstrated satisfactory psychometric properties and is comparable to other reliable scales.^22^^,^^23^ 

The DASS-21 is the short-form version of the original self-reported 42-item questionnaire and has demonstrated good to excellent internal consistency,^24^adequate reliability and construct validity.^25^ It includes three self-reported scales designed to measure the negative emotional states of depression, anxiety and stress. Each of the three scales contains 7 items scored on a Likert scale from 0-3 (0: Did not apply to me at all, 1: Applied to me to some degree or some of the time, 2: Applied to me to a considerable degree or a good part of the time, 3: Applied to me very much or most of the time). Depression, anxiety and stress scores are calculated by summing the scores of the relevant items. Because the DASS-21 is a short-form version of the DASS (42 items), the final score for each sub-scale is multiplied by two and evaluated according to its severity rating index. The questionnaire was piloted with 28 students from other health colleges, and the language was simplified based on the feedback received.

**Table 2:** Levels of depression, anxiety and stress among study participants

Stress N (%)	Anxiety N (%)	Depression N (%)	Levels
112 (45.3)	82 (33.2)	109 (44.1)	Normal
36 (14.6)	28 (11.3)	35 (14.2)	Mild
49 (19.8)	53 (21.4)	53 (21.4)	Moderate
30 (12.1)	22 (8.9)	21 (8.5)	Severe
20 (8.1)	62 (25.1)	29 (11.7)	Extremely severe

### Data collection methods and procedures

Class leaders were contacted, and a 10-minute meeting after a lecture was arranged for each student year. One of the authors briefly explained the project, and hard copies of the questionnaires were then distributed. Participation was voluntary, the purpose of the research was stated on the first page of the questionnaire, confidentiality and anonymity were assured, and written informed consent was obtained from all participants.    

### Statistical analysis

 The statistical analysis was performed using Statistical Package for Social Sciences software (SPSS version 20) (IBM Corporation, New York, USA). Descriptive statistics (means, standard deviations, percentages and frequencies) were calculated to assess the percentages and levels of depression, anxiety and stress among the study participants. Multiple linear regression was used to test whether depression, anxiety and stress could be predicted by the different variables in the questionnaire.

## Results

### Percentages of depression, anxiety, and stress and DASS scores

Abnormal levels of depression, anxiety and stress were observed in 55.9%, 66.8% and 54.7% of the respondents, respectively (Table 2). The mean total scores for the respondents were 12.79 (SD = 10.73) for depression, 12.35 (SD = 9.48) for anxiety and 17.17 (SD = 10.02) for stress. Alarmingly, severe and extremely severe scores for depression, anxiety, and stress were reported in 20.2%, 34.0% and 20.2% of students, respectively.

### DASS scores and student factors

The Cronbach’s alphas for each of the 7-item subscales (Depression = .889, Anxiety = .822, Stress = .865) indicated the high internal consistency and reliability of the questionnaire. The residual plot distribution revealed a slight positive skew towards depression (K-S test, p = .042; skewness = .654) and anxiety (K-S test, p = .251) but no marked inflation due to collinearity.

### Depression

A significant regression equation was found (R^2^_Adjusted_ = .129. F_(__9,237)_ = 5.044, p < .001). Satisfaction with relationships with peers significantly predicted depression (b = -3.527, t_(__237)_ = -3.592, p < .001), as did satisfaction with relationships with college faculty (b = -2.318, t_(237)_ = -2.709, p = .007). Depression decreased when satisfaction with peer and faculty relationships increased. Low satisfaction with relationships with peers was a stronger predictor of depression (β = -.229) than low satisfaction with relationships with faculty (β = -.174) (Table 3).

### Anxiety

A significant regression equation was found (R^2^_Adjusted_ = .123, F_(__9,237)_ = 4.825, p < .001). Gender significantly predicted anxiety (b = -3.589, t_(__237)_ = -2.424, p = .016), as did satisfaction with relationships with peers (b = -2.119, t_(237)_ = -2.432, p = .016) and satisfaction with relationships with faculty (b = -2.213, t_(237)_ = -2.914, p = .004). Anxiety decreased when there was an increase in satisfaction with peer and faculty relationships and when the student was male. Gender was a slightly stronger predictor of anxiety (β = -.189) than low satisfaction with relationships with faculty (β = -.188), while low satisfaction with relationships with peers (β = -.155) was the weakest predictor (Table 3).

### Stress

A significant regression equation was found (R^2^_Adjusted _= .163, F_(__9,237)_ = 6.305, p < .001). Gender significantly predicted stress (b = -4.099, t_(237)_ = -2.683, p = .008), as did dentistry as the student’s first choice of study (b = -2.648, t_(237)_ = -2.011, p = .045), satisfaction with relationships with peers (b = -2.096, t_(237)_ = -2.331, p = .021), and satisfaction with relationships with faculty (b = -2.854, t_(237)_ = -3.642, p < .001). Stress decreased when (a) the student was male; (b) there was an increase in satisfaction with relationships with peers; (c) there was an increase in satisfaction with relationships with college faculty; or (d) dentistry was the student’s first choice of study. The strongest predictor of stress was low satisfaction with faculty relationships (β = -.229), followed by gender (β = -.204) and low satisfaction with relationships with peers (β = -.145), and the weakest predictor was when dentistry was not the student’s first choice of study (β = -.127) (Table 3).

**Table 3:** Multiple linear regression model for the prediction of depression, anxiety and stress in dental students

Predictor	Subscale	Unstandardized Coefficients		Standardized Coefficients	t	p	Collinearity Statistics	
		b	SE	β			Tolerance	VIF
Constant	D	33.597	8.272		4.062	.000^*^		
	A	22.598	5.559		4.065	.000^*^		
	S	29.473	5.737		5.137	<.001^*^		
Gender (1 = Male, 0 = Female)	D	-2.275	1.668	-.106	-1.364	.174	.586	1.705
	A	-3.589	1.480	-.189	-2.424	.016^*^	.586	1.705
	S	-4.099	1.528	-.204	-2.683	.008^*^	.586	1.705
Marital status (1 = Married, 0 = Not Married)	D	0.324	2.310	.009	0.140	.889	.937	1.067
	A	3.331	2.049	.100	1.625	.105	.937	1.067
	S	2.762	2.115	.079	1.306	.193	.937	1.067
Year of study (1 to 5)	D	0.880	.496	.117	1.774	.077	.821	1.218
	A	3.331	2.049	.100	1.625	.105	.937	1.067
	S	0.573	.454	.081	1.261	.209	.821	1.218
GPA of the previous year (2 to 4)	D	0.893	1.221	.058	0.731	.465	.569	1.758
	A	3.331	2.049	.100	1.625	.105	.937	1.067
	S	1.318	1.118	.091	1.179	.240	.569	1.758
Was studying dentistry your first choice? (1 = Yes, 0 = No)	D	-1.640	1.438	-.074	-1.140	.255	.847	1.181
	A	-2.430	1.276	-.124	-1.904	.058	.847	1.181
	S	-2.648	1.317	-.127	-2.011	.045^*^	.847	1.181
Do you have financial responsibilities towards your family? (1=Yes, 0 = No)	D	-0.401	1.800	-.014	-0.223	.824	.887	1.127
	A	-2.430	1.276	-.124	-1.904	.058	.847	1.181
	S	-0.756	1.648	-.028	-.459	.647	.887	1.127
How satisfied are you with your relationship with your peers? (1 to 3)	D	-3.527	.982	-.229	-3.592	<.001^*^	.875	1.143
	A	-2.119	.871	-.155	-2.432	.016^*^	.875	1.143
	S	-2.096	.899	-.145	-2.331	.021^*^	.875	1.143
How satisfied are you with your relationship with college faculty? (1 to 3)	D	-2.318	.856	-.174	-2.709	.007^*^	.859	1.165
	A	-2.213	.759	-.188	-2.914	.004^*^	.859	1.165
	S	-2.854	.784	-.229	-3.642	<.001^*^	.859	1.165
Overall, are you satisfied with your experience at college?(1 to 3)	D	-0.433	.921	-.029	-0.470	.639	.923	1.084
	A	0.793	.817	.060	.971	.333	.923	1.084
	S	-0.017	.843	-.001	-.020	.984	.923	1.084

^*^Significant predictor (p < .05)

D=depression, A=anxiety, S=stress, SE=standard error, VIF=variance inflation factor

### Stress coping mechanisms

**Figure 1:**
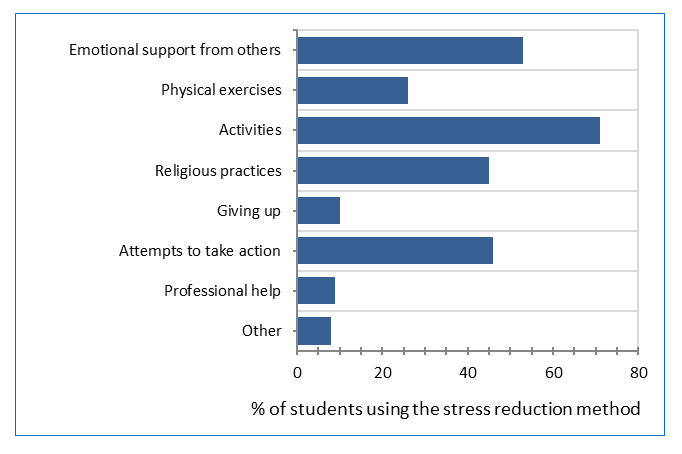
Students’ methods of stress reduction

The most frequently mentioned coping method for relieving stress was "activities such as watching television, reading, sleeping and shopping" (71%), followed by "emotional support from others" (53%). Under “other” mechanisms, eating, traveling and smoking were mentioned by some students as stress coping methods. Two students mentioned using recreational drugs to relieve stress (Figure 1).

## Discussion

Undergraduate dental education in Saudi Arabia is a seven-year program. The first year is a general preparatory year that is common to all health colleges, followed by 5 years of dental school. The final year is a 12-month internship program. The first year consists mainly of courses in basic, medical, and dental science. Students enroll in dental school directly from high school; the level of competition is very high, and only the best students with the highest grades and performance indicators at school and on admission exams and interviews are accepted. This competition persists throughout dental education, as high grades and above-average performance are expected and represent the norm. Additional pressure stems from the large amount of new information that students must learn and the technical skills they must master. Schmitter and colleagues^26^ noted that dental education is more stressful than even medical education.

In this study, the occurrence of depression, anxiety, and stress among dental students was high, and the levels of these conditions were abnormal in more than half the students. The figures in this study were comparable to those of Aboalshamat and colleagues^27^ in a sample of medical and dental students. Additionally, this observation is consistent with the findings of previous studies investigating stress among dental students in Saudi Arabia^6^^,^^27^^,^^28^ and other countries^18^^,^^29^^-^^32^ and with several studies investigating depression^16^^,^^19^^,^^20^ and anxiety^19^^,^^31^ in dental students. Dental students report higher levels of anxiety, depression, obsessive-compulsive disorders, and interpersonal sensitivity than the general population and age-matched students in other fields of study.^2^^,^^33^

The higher rate of and scores for anxiety (compared with depression and stress) confirm the observation that anxiety has become the most common mental health problem among college students.^34^

The high levels of depression, anxiety and stress could be attributed to the pressure exerted on students during dental education by their workload, clinical requirements, examinations and grades.^4^^,^^8^ Rosal and colleagues^35^ noted that when medical students enter medical school, they exhibit depression levels comparable to those of the general population, but their levels of depression increase significantly during medical school. In fact, dental students report more psychological problems than medical students,^19^with levels of depression that are sometimes three times as high.^16^

Female students universally report higher levels of stress and anxiety,^2^^,^^4^^,^^6^^,^^18^-^20^^,^^28^^,^^29^^,^^36^ although there are a few exceptions.^17^^,^^37^ This observation could be explained by the intrinsic psychological differences between genders: females are more likely to articulate their worries and emotions. Students’ first choice for field of study was also a significant predictor of stress levels, and students whose first choice was dentistry reported less stress.^2^^,^^4^^,^^6^^,^^17^^,^^18^^,^^29^^,^^38^ In KSU, admittance into dental school is based on students’ GPA during the preparatory year. Students may experience dissatisfaction studying for a career in which they are not interested; consequently, students should not be admitted to a training program that is not their first choice.

Marital status and financial responsibilities were not significant predictors of depression, anxiety, or stress in the current research. Other studies have also found that students’ marital status is not a significant predictor.^6^^,^^39^ Although married students may be more prone to stress due to their increased responsibilities, the marital relationship and care of children may be a source of support. The lack of tuition fees for education at KSU in addition to the monthly stipend may minimize the effects of financial factors on students' perceived levels of depression, anxiety and stress. This finding is in agreement with previous results that demonstrated no association between students' financial aid and depression^16^ or stress levels.^10^

Study year and academic achievement (measured by GPA in this study) are the most commonly reported academic factors that significantly affect dental students’ psychological health. Studies have reported higher stress levels among students with the lowest GPAs and among students with average GPAs than among other students.^4^^,^^6^^,^^10^ Interestingly, both factors were non-significant predictors of all the subscales in this study. Sanders and Lushington^37^ also noted that high levels of stress do not result in lower academic performance and suggested that the dental education environment itself may be a cause of stress rather than grades alone. Although several investigations have reported that senior dental students or students transitioning from preclinical to clinical training exhibit the most stress,^4^^,^^6^^,^^28^ year of study has not been a significant factor in other studies.^40^^,^^41^ Our findings could indicate that all students experience immense pressure and stress regardless of their level of study. Moreover, prior reports have used a different tool, the DES questionnaire, which seeks to identify stressors, whereas the DASS used in the current study tends to account for the physical and psychological symptoms of stress. This view is supported by the findings of Peker and colleagues,^17^ who reported that levels of stress were significantly higher in 4th-year than in 1st-year students when measured by the DES but that levels of depression did not significantly differ when measured by the BDI. This finding is consistent with the results of studies utilizing the Psychological General Well-Being Index.^2^^,^^19^^,^^42^ Dental students experience many stressors throughout their education, and the DES identifies additional stressors that arise as students progress through dental school, such as patient factors and worries about the future and job opportunities. Although the types of stressors may vary by year,^6^^,^^28^^,^^43^ the stressors experienced in different years may have comparable effects on students’ physical and psychological symptoms. 

The most significant and strongest predictors in this study were students’ satisfaction with their faculty and peer relationships, followed by their overall experience at the college. Therefore, factors related to human relationships appear to have a greater impact on psychological health than other academic factors. Similarly, Wayt^44^ observed that personal relationships have a greater effect on college students’ persistence in studying than academic factors.

Students’ satisfaction with their learning environment has been discussed in the literature. In previous studies, students have either been unsatisfied with faculty support^45^ or have perceived faculty members and administrators as a significant source of stress.^2^^,^^4^^,^^6^^,^^8^^,^^37^^,^^43^^,^^46^ Our findings revealed that this dissatisfaction leads to higher levels of stress, anxiety and depression. Faculty support has been associated with significantly lower stress levels in dental students. Similarly, students have stated that their relationships with faculty or staff critically affect their learning experience,^37^^,^^46^^,^^47^ development,^48^ and grades.^37^ Therefore, a humanistic learning environment is encouraged, in which students can learn freely without intimidation and through close professional relationships with faculty fostered by mentoring, advising, and group interaction.^49^^,^^50^ Al-Mohaimeed and Khan^51^ surveyed medical students regarding the qualities of effective teachers and found that the students valued expertise, organization of good lectures, respect and ability to relate to students more highly than disposition to give high grades. 

Previous studies have focused more on students’ relationships with staff than their relationships with fellow students. In this study, we found that one of the strongest predictors of stress was peer relationships; satisfactory peer relationships protected against distress. Previous studies have reported that the higher students’ social support is, the lower their symptoms of stress.^52^^,^^53^ Furthermore, when peers act as mentors, stress and anxiety are significantly reduced.^54^

Stressed individuals typically adopt certain coping patterns as a natural response to stress and to alleviate the associated psychological tension. In our study, the majority of the students actively engaged in activities unrelated to the profession of dentistry to relieve stress. Previous studies have found that the most common coping method is emotional support from others, specifically, talking to friends and family.^30^^,^^55^ When students lack this source of social support, levels of stress increase.^56^ Smoking was one of the stress relievers mentioned by students in the current study, and in a recent study involving dental students at KSU, stress was found to be the main reason for smoking, reported by approximately half the students who smoked.^57^ A less frequently mentioned but more serious source of stress relief was recreational drug use. Prinz and colleagues^19^ reported that students with the highest levels of depression and anxiety exhibit higher degrees of dysfunctional coping. These adverse coping patterns could have potentially serious effects on both physical and mental health and should not be underestimated.

Research has also suggested that students with certain personality traits are more prone to distress. Specifically, the risk of depression may increase with maladaptive perfectionism,^58^ type A personality, and anger suppression,^36^ whereas self-actualization, self-awareness, and a sense of fulfillment may lower the risk of depression.^59^^,^^60^ Emotional intelligence has been found to both protect against perceived stress^38^ and affect coping with stress. Dental students with higher levels of emotional intelligence adopt reflection and appraisal as well as social and interpersonal coping methods. Students with low emotional intelligence scores engage in habits to cope with stress that damage their health.^61^ Zhou and colleagues^62^ further noted that perceived social support protects perfectionist students from experiencing depression or anxiety.

Several strategies for stress management among dental students have been introduced and discussed in the literature, including relaxation strategies, interpersonal approaches such as counseling systems, programs designed to improve studying and test-taking skills and stress management workshops.^63^ Because stress in the dental educational environment is typically unavoidable, stress management strategies can be recommended as an early and integral part of the dental curriculum. These strategies could focus mainly on improving the perception of stressful situations, the development of coping skills and the avoidance of maladaptive coping. Additionally, more emphasis should be placed on the importance of humanistic faculty-student relationships. The current study site has implemented several positive changes to improve the learning environment for students, including establishing a student council, obtaining student feedback on faculty and courses, creating a student support unit, and assigning academic advisors to each student throughout his or her education. It is also suggested that students be allowed to choose the clinical instructors with whom they feel most comfortable working. The student-to-instructor ratio could also be reduced to promote more active student involvement and to allow for more effective feedback. Additionally, students can be trained to foster emotional intelligence and organizational and time-management skills,^61^^,^^64^ and students may benefit from training to improve their communication skills in order to facilitate interactions with faculty and administrators.^46^

Although this study has several strengths, such as the excellent response rate, random sampling method, and the simple and concise test tool, it also has some limitations. The cross-sectional design does not allow for assessments of changes in psychological status over time. In addition, given the self-reported nature of the assessment tool, we cannot rule out response bias. Furthermore, our literature review was limited to articles published in English, which may preclude comparisons with significant findings reported in other languages. To improve the accuracy of the outcomes, studies using a longitudinal design that include more schools at the national or international level are recommended for future research.  

## Conclusions

This study aimed to assess the levels of depression, anxiety and stress in undergraduate dental students and found that despite excluding participants currently undergoing psychological management, the levels of these conditions were relatively high. The actual numbers may be even higher than those reported herein. Participants with abnormal depression and anxiety scores require a clinical diagnosis to receive prompt treatment. Furthermore, to ensure early identification of and intervention for psychological conditions, both students and faculty should be educated regarding the physical and psychological signs and symptoms of anxiety and depression. More attention should be devoted to susceptible individuals, such as female students, with the aim of improving relationships and satisfaction levels. Strategies for stress prevention and management should be implemented in dental schools to improve students’ well-being, prevent drop out and ensure proper patient care. The persistence of these problems may lead to further physical and psychological complications that could continue after graduation, resulting in unhealthy dentists or early retirement and thereby affecting both the quantity and quality of the workforce.

### Acknowledgments

The authors would like to acknowledge the efforts of Dr Manal Alkadi, who had an important role and contributed to this study but humbly chose not to share co-authorship of this article.

### Conflict of Interest

The authors declare that they have no conflict of interest.
